# Molecular Epidemiology and Evolution of Human Enterovirus Serotype 68 in Thailand, 2006–2011

**DOI:** 10.1371/journal.pone.0035190

**Published:** 2012-05-07

**Authors:** Piyada Linsuwanon, Jiratchaya Puenpa, Kamol Suwannakarn, Vittawat Auksornkitti, Preeyaporn Vichiwattana, Sumeth Korkong, Apiradee Theamboonlers, Yong Poovorawan

**Affiliations:** Chulalongkorn University, Bangkok, Thailand; University of Illinois at Chicago, United States of America

## Abstract

**Background:**

Publications worldwide have reported on the re-occurrence of human enterovirus 68 (EV68), a rarely detected pathogen usually causing respiratory illness. However, epidemiological data regarding this virus in particular on the Asian continent has so far been limited.

**Methodology/Findings:**

We investigated the epidemiology and genetic variability of EV68 infection among Thai children with respiratory illnesses from 2006–2011 (n = 1810). Semi-nested PCR using primer sets for amplification of the 5′-untranslated region through VP2 was performed for rhino-enterovirus detection. Altogether, 25 cases were confirmed as EV68 infection indicating a prevalence of 1.4% in the entire study population. Interestingly, the majority of samples were children aged >5 years (64%). Also, co-infection with other viruses was found in 28%, while pandemic H1N1 influenza/2009 virus was the most common co-infection. Of EV68-positive patients, 36% required hospitalizations with the common clinical presentations of fever, cough, dyspnea, and wheezing. The present study has shown that EV68 was extremely rare until 2009 (0.9%). An increasing annual prevalence was found in 2010 (1.6%) with the highest detection frequency in 2011 (4.3%). Based on analysis of the VP1 gene, the evolutionary rate of EV68 was estimated at 4.93×10^−3^ substitutions/site/year. Major bifurcation of the currently circulating EV68 strains occurred 66 years ago (1945.31 with (1925.95–1960.46)95% HPD). Among the current lineages, 3 clusters of EV68 were categorized based on the different molecular signatures in the BC and DE loops of VP1 combined with high posterior probability values. Each cluster has branched off from their common ancestor at least 36 years ago (1975.78 with (1946.13–1984.97)95% HPD).

**Conclusion:**

Differences in epidemiological characteristic and seasonal profile of EV68 have been found in this study. Results from Bayesian phylogenetic investigations also revealed that EV68 should be recognized as a genetically diverse virus with a substitution rate identical to that of enterovirus 71 genotype B (4.2×10^−3 ^s/s/y).

## Introduction

Human enterovirus 68 (EV68) is a sporadically detected viral pathogen associated with respiratory tract illnesses. EV68 belongs to the family *Picornaviridae* which contains socially and economically important pathogenic viruses such as foot-and-mouth disease virus, human enterovirus, rhinovirus, and hepatitis A virus. It has been classified in the genus *Enterovirus* species D which currently comprises 2 important human pathogens, EV serotype 70 and serotype 94. It possesses a positive single-stranded RNA genome of ∼7.5 kb in length encased by a highly structured icosahedral capsid. The viral genome is composed of a 5′-untranslated region (5′UTR) followed by a long single open reading frame and terminated by a short 3′UTR with genetically encoded poly-A tract. The coding sequence is co-translationally processed by a virus encoded protease to yield 4 structural (viral capsid protein (VP)-1 to VP4) and 7 nonstructural proteins (2A to 2C and 3A to 3D) which are responsible for viral replication, protein processing and also contribute to shutting down the host cell’s protein production.

Many methods have been established for EV typing. Normally, traditional methods rely on biological properties of the viruses such as antigen distinction which subdivides EV into ‘serotypes’. However, this method is time consuming, expensive, and could not classify some newly identified EVs. Consequently, as alternative and sensitive methods of classification of all EVs, reverse transcription polymerase chain reaction (RT-PCR) and nucleotide sequencing of the entire genome or partial VP4 or VP4/VP2 regions combined with the results from VP1 gene have recently been utilized for characterizing EVs [1]–[4]. EV68 was originally isolated in California in 1962 from children with pneumonia and bronchiolitis by using virus isolation and sero-neutralization approaches [5]. In contrast to other EVs, EV68 shares common biological properties with human rhinovirus (RV), which is acid sensitive virus and grow efficiently at an optimum temperature of ∼33°C [6], [7]. The virus is usually found predominantly in respiratory illness patients. Infection by this virus can cause various disease severities ranging from mild respiratory illnesses such as common cold to severe acute lower respiratory tract infections (ALRTI) including pneumonia, wheezing and bronchiolitis [8]–[11]. Three fatal cases caused by severe respiratory illnesses and associated with EV68 infection have been reported [10], [11]. Recent studies have suggested that asthmatic individuals infected with EV68 have a propensity to develop unstable asthma or an acute attack [9]. Despite the medical burden of the disease, no effective antiviral therapies have been approved for either prevention or treatment of EV68 infection. Awareness of its precise global distribution, longitudinal epidemiological profiles, its pathogenic role, and evolutionary history of EV68 are also still lacking. Furthermore, since the re-occurrence of EV68 infection in several countries worldwide [8], only 3 epidemiological studies of EV68 have been reported from the Asian continent (2 from Japan during the study periods of 2006–2009 and 2009–2010 and 1 from the Philippines during 2009–2010) [9]–[11].

To address these concerns, we conducted a prospective longitudinal population-based study among Thai children aged 15 years (yrs.) and below with acute respiratory tract illness (ARTI) symptoms during 2006–2011. Semi-nested PCR amplification covering a highly conserved 5′UTR up to and including the 5′ terminus of the capsid VP2 gene (5′UTR/VP2) and nucleotide sequencing were applied to elucidate many aspects of the epidemiology and evolution of the virus. The primary objective was to determine whether EV68 infection was associated with ARTI among a large number of children in Thailand. The secondary objective was to better understand the evolutionary history of EV68 circulating lineages and to determine how evolution of this virus might have been shaped by its population dynamics.

## Results

### Study Population

We retrospectively analyzed 1822 stored respiratory specimens obtained from 1785 children with ARTI complications who visited several hospitals located in Bangkok, Thailand between February 14, 2006 and November 8, 2011. The main characteristics of these populations are summarized in Table 1. Of these eligible specimens, 24 originated from 12 children and collected in the course of the same hospitalization for treatment follow-up. Twenty-five children had multiple hospitalizations; 22 had 2 admissions and 3 had 3 admissions. Therefore, a total of 1810 respiratory specimens were available for testing.

**Table 1:** Main characteristics of the study populations and respiratory specimens.

Years of study	Sample	N	Infant	Pre-school	Primary school	Secondary school
Feb 2006–Jul 2008	NP	383	318 (0.9+0.5)	42 (3.5+0.7)	14 (8.9+2.1)	4 (13.5+0.5)
Jun 2009–Sep 2011	NS	1213	238 (1.5+0.6)	352 (4.3+0.8)	442 (8.7+1.9)	167 (14.6+0.9)
Jul 2010–Nov 2011	NP	214	153 (1.1+0.7)	26 (3.7+0.9)	24 (8.5+1.9)	4 (13.3+0.4)
Total		1810	709	420	480	175
Patient age, yrs			1.1+0.6	3.3+0.7	8.7+1.9	14+0.9

NP, Nasopharyngeal aspiration specimens; NS, Posterior oropharyngeal and nasal swab specimens.

Patient age was showed by using mean ± standard deviation.

To assess the distribution of respiratory virus infection with regard to the specific children’s age and severity of respiratory illness, the subjects were categorized into 4 groups as follows: infant (≤2 yrs.), pre-school (>2 to 5 yrs.), primary-school (>5 to 12 yrs.), and secondary school children (>12 to 15 yrs.). Of 1810 cases, 26 cases (1.4%; 14 outpatients and 12 hospitalized patients) could not provide specific information with regards to age, 39.2% were infants (n = 709), 23.2% were pre-school (n = 420), 26.5% were primary-school (n = 480), and 9.7% were secondary school children (n = 175) (Table 1). The age distribution of enrolled patients was between 1 day (d.) to 15 yrs. Clinical severity of ARTI cases was defined as follows: pediatric patient with ARTI complications not requiring hospitalization and O_2_ supplementation and normal to slightly increased respiratory rate was defined as ‘mild’ case, patient with ARTI symptoms, requiring hospitalization, and either did or did not require supplemental O_2_ was defined as ‘moderate’ case, patient with severe respiratory failure with O_2_ saturation <90%, requiring medical ventilation or O_2_ supplementation, and admission to the Intensive Care Unit (ICU) was defined as ‘severe’ case. Consequently, 32.1% (n = 581), and 0.9% (n = 16) of the entire study population were considered as moderate and severe cases, respectively, while the remaining cases were mild ARTIs (67%; n = 1213).

### Detection of EV68 in ARTI Patients

**Figure 1:**
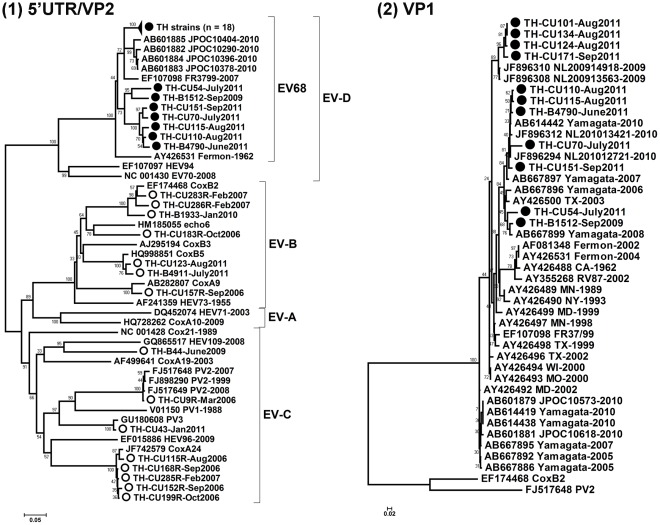
Neighbor-joining (NJ) estimate of phylogenetic relationship for human enteroviruses detected among Thai pediatric children during 2006–2011. The NJ trees were constructed from nucleotide alignments of (A) the 5′UTR/VP2 (612 nt) and (B) partial VP1 region (257 nt) using MEGA software. The genetic distances were calculated according to the Kimura-parameter model. Bootstrap support values with 1000 pseudo-replicates were plotted at the internal branch nodes. EV species A to D were labeled. Period of the sample collection was plotted for each lineage. EV68 and other EVs identified in this study (TH-strain) are indicated in black and white circles, respectively. Virus sequence names or location of isolations shown with GenBank accession numbers were reference strains. Scale bar indicates number of nucleotide substitutions per site.

Of children with ARTI complications, 812 were infected by at least 1 respiratory virus accounting for 44.9% of all enrolled cases. Rhino-enterovirus (RV-EV) was detected in 15.9% (288/1810). Among these, 25 were confirmed as EV68 infection accounting for 8.7% of RV-EV positive specimens and indicating a prevalence of 1.4% in the entire study population. To investigate the relationship between EV68 characterized in Thailand (EV68-TH strains) and the previously defined strains, phylogenetic tree of the 5′UTR/VP2 region (Fig 1A) and their sequence identity matrix (Table 2) were constructed. The 5′UTR/VP2 region of EV68-TH strains exhibited 86.3–97.1% nucleotide identities to each other. EV68-TH strains shared 71–84.1% nucleotide identity with the first identified strain ‘Fermon’ (AY426531) and 67.7–93.7% with those of strains identified from other countries. All EV68 infected cases were confirmed by amplification of the VP1 gene. Despite performing semi-nested PCR and nested-PCR amplifications yielding a 2^nd^ PCR product of ∼850, 450 or 250 nucleotides (nt), only 11/25 EV68-positive specimens could be amplified. Fig 1B shows the phylogenetic tree of the capsid VP1 encoding gene. Comparisons of the nucleotide sequence of VP1 revealed 84.4–100% identity among EV68-TH strains and 81.7–92.2% identity between TH-strains and the primary strains. The TH-strains shared 85.6–99.8% nucleotide identity with the EV68 strains currently circulating or identified between 2008 and 2010. Demographic data and clinical characteristics of these 25 EV68-positive children are summarized in Table 3.

**Table 2:** Nucleotide identity matrix obtained for the alignment of 5UTR′/VP2 region of EV68 identified from Thailand and reference strains of EV68 and other members of enterovirus species D (EV94 and EV70).

Sequence	CU134	CU54	B2054	B2060	B1512	B196	EV94	JPOC	FR3799	Fermon
CU54	86.2									
B2054	99.6	85.8								
B2060	99.8	86.0	99.4							
B1512	88.0	92.3	87.7	87.9						
B196	99.1	85.3	98.7	98.9	87.5					
EV94	68.6	70.7	68.6	68.4	69.6	67.9				
JPOC	93.5	86.9	93.2	93.4	88.7	93.0	67.7			
FR3799	93.9	89.7	93.5	93.7	91.8	93.0	68.6	94.6		
Fermon	82.9	82.8	82.6	82.8	84.1	82.4	71.0	82.9	82.9	
EV70	71.8	70.8	71.8	71.6	70.1	70.9	75.2	69.1	69.6	70.7

The GenBank accession numbers of the previously published sequences are as follows: EV94, EF107097; EV70, NC_001430; JPOC, AB601884; FR3799, EF107098; Fermon, AY426531.

**Table 3:** Clinical characteristics and demographic data of the 25 cases of EV68 infection.

Strain	Sample	Date	Age	Sex	Co-infection	Diagnosis	5′UTR/VP2	VP1
TH-B196	NS	30/6/2009	13	M	pH1N1/2009	ILI	JQ411799	N/A
TH-B211	NS	1/7/2009	14	F	pH1N1/2009	ILI	JQ411798	N/A
TH-B323	NS	4/7/2009	11	F	pH1N1/2009	ILI	JQ411797	N/A
TH-B521	NS	9/7/2009	10	M	pH1N1/2009	ILI	JQ411796	N/A
TH-B1512	NS	2/9/2009	15	F	–	ILI	JQ411794	JQ411802
TH-B2054	NS	9/2/2010	12	M	pH1N1/2009	ILI	JQ411791	N/A
TH-B2060	NS	10/2/2010	8	F	–	ILI	JQ411792	N/A
TH-B2114	NS	16/2/2010	9	M	–	ILI	JQ411789	N/A
TH-B2192	NS	24/2/2010	9	F	–	ILI	JQ411793	N/A
TH-B2213	NS	26/2/2010	8	F	–	ILI	JQ411790	N/A
TH-B2303	NS	12/3/2010	14	M	–	ILI	JQ411786	N/A
TH-B2340	NS	1/4/2010	6	M	–	ILI	JQ411788	N/A
TH-B2370	NS	2/6/2010	14	M	Flu-B	ILI	JQ411787	N/A
TH-B3611	NS	15/9/2010	15	F	Flu-B	ILI	JQ411781	N/A
TH-B4105	NS	5/10/2010	6	F	Flu-B	ILI	JQ411782	N/A
TH-B4790	NS	27/6/2011	1	F	–	ILI	JQ411785	JQ411809
TH-CU54	NP	8/7/2011	2	F	–	VP	JQ411783	JQ411805
TH-CU70	NP	25/7/2011	1	F	–	VP	JQ411784	JQ411801
TH-CU101	NP	15/8/2011	2	F	–	VP	JQ411776	JQ411804
TH-CU110	NP	18/8/2011	2	F	RSV-A	VP	JQ411777	JQ411810
TH-CU115	NP	22/8/2011	4	F	–	VP	JQ411778	JQ411811
TH-CU124	NP	26/8/2011	7	F	–	VP	JQ411779	JQ411807
TH-CU134	NP	31/8/2011	2	M	–	VP	JQ411775	JQ411803
TH-CU151	NP	12/9/2011	4	M	–	VP	JQ411780	JQ411806
TH-CU171	NP	23/9/2011	7 m	M	–	VP	JQ411795	JQ411808

pH1N1/2009, pandemic influenza A virus subtype H1N1/2009; Flu-B, Influenza B virus; RSV, Respiratory syncytial virus; ILI, influenza like illness; VP, Viral pneumonia.

Results from RV-EV specific PCR amplification and nucleotide sequencing also revealed that 15 specimens had other EV infections (Fig 1A). Of these, 1 specimen was classified as poliovirus Sabin-2 with similar proportions of poliovirus Sabin-3 (vaccine strains confirmed), coxsackie virus (CA)-A9, echovirus 9, echovirus 6, and EV109 whereas CA-B5 and CA-B2 were identified in 2 samples each. Five samples were positive for CA-A24. The overall frequency of detection was 0.8% (15/1810).

### Clinical Manifestations of EV68 Infected Patients

EV68 were detected as a sole pathogen in 16/25 cases (64%). Co-infections by other respiratory viruses were found in 9 cases (36%) including 1 with respiratory syncytial virus (RSV) type A, 3 with influenza B virus (Flu-B), and the remaining 5 patients were co-infected with pandemic H1N1/2009 virus (pH1N1/2009). As shown in Table 4, age specifics of children with ARTI complications were 1% infant (7 cases; 1 outpatient and 6 hospitalized patients), 2% pre-school (2 cases; all were hospitalized cases), 2.1% primary-school (10 cases; 9 outpatients and 1 hospitalized patient), and 3.4% secondary school children (6 cases; all were outpatients). The average age of EV68-associated ARTI cases was statistically higher than that of the non-infected group (7.6+5.0 yrs. vs 5.0+4.4 yrs.; *p* = 0.04). Without co-infection by other pathogens, the average age of EV68-associated ARTIs was 5.8+4.4 yrs. The majority of EV68 cases were primary school (40% of EV68 cases and 2.1% of primary school cases) and secondary-school children (24% of EV68 cases and 3.4% of secondary-school cases). Of EV68-positive children, 64% were children aged >5 yrs. (*p* = 0.016). Female-to-male ratio of EV68 infected patients was 1.5∶1. As for the contribution of the virus to disease severity, the majority of positive cases had moderate severity (32% (8/25)). One patient presented with severe clinical characteristics and required admission to the ICU. EV68-associated ARTI represented 6.3% (1/16) of all severe cases. None of the patients died. During the study period, hospitalized patients with ARTI were more likely to be infected with EV68 compared to outpatients (1.5% (9/597) vs 1.3% (16/1213)) but this difference did not show any statistical significance. Summary of discharge diagnoses of EV68-confirmed cases included viral pneumonia (n = 8), asthma (n = 1), and influenza-like illness (ILI) (n = 16). Clinical manifestations of the 9 hospitalized patients with EV68 infections are summarized in table 5. Supplemental O_2_ and medical ventilation were required in all EV68-hospitalized cases. The most common clinical characteristics of these patients were fever, cough and dyspnea (77.8%), and wheezing (66.7%). Hospitalization lasted a median of 5 d (ranging from 3–16 d). Four patients with EV68 infections had no medical history while 3 had underlying disease such as gastro-esophageal reflux, congenital heart disease, and autism and 2 had a history of asthma. One of these had RSV-A co-infection in the nasal aspiration specimen. However, there was no significant difference in disease severity between the case with and without RSV co-infection.

**Table 4:** ARTI cases infected by EV68 in Thailand from June 2009 to November 2011.

Group	Mild ARTI	Moderate ARTI	Severe ARTI	EV68 cases	Average age
Infant (n = 709)	1 (0.1%)	5 (0.9%)	1 (6.3%)	7 (1%)	1.5+0.7
Pre-school (n = 420)	0	2 (0.3%)	0	2 (0.5%)	4
Primary-school (n = 480)	9 (0.7%)	1 (0.2%)	0	10 (2.1%)	8.6+2.0
Secondary-school (n = 175)	6 (0.5%)	0	0	6 (3.4%)	14.2+0.8
Total	16 (1.3%)	8 (1.4%)	1 (6.3%)	25	

Average age = mean ± standard deviation.

**Table 5:** Summary of clinical manifestations and diagnoses of the 9 patients hospitalized with EV68 infection.

EV68 strain	LT	Underlying	Symptom	O_2_ therapy
TH-CU54	5	No	Fever, cough, dyspnea, wheezing	Yes
TH-CU70	3	No	Fever, cough, dyspnea, wheezing	Yes
TH-CU101	5	Autism	Fever, cough, vomiting	Yes
TH-CU110	4	No	Fever, cough, vomiting, diarrhea, dyspnea, wheezing	No
TH-CU115^a^	4	No	Tachypnea, wheezing, runny nose	Yes
TH-CU124^a^	7	No	Fever, cough, dyspnea, wheezing	Yes
TH-CU134	8	GER	Fever, cough, dyspnea, wheezing	Yes
TH-CU151	3	No	Fever, cough, runny nose, dyspnea, wheezing	Yes
TH-CU171^b^	16	CHD	cough, dyspnea, upper airway obstruction	Yes

a  =  had history of asthma/b  =  admitted to the intensive care unit.

All of these EV68 infected patients required nebulizer.

LT, Length of hospitalization (days); GRE, Gastro-esophageal reflux; CHD, Congenital heart disease.

### Seasonality of EV68 Infections

**Figure 2:**
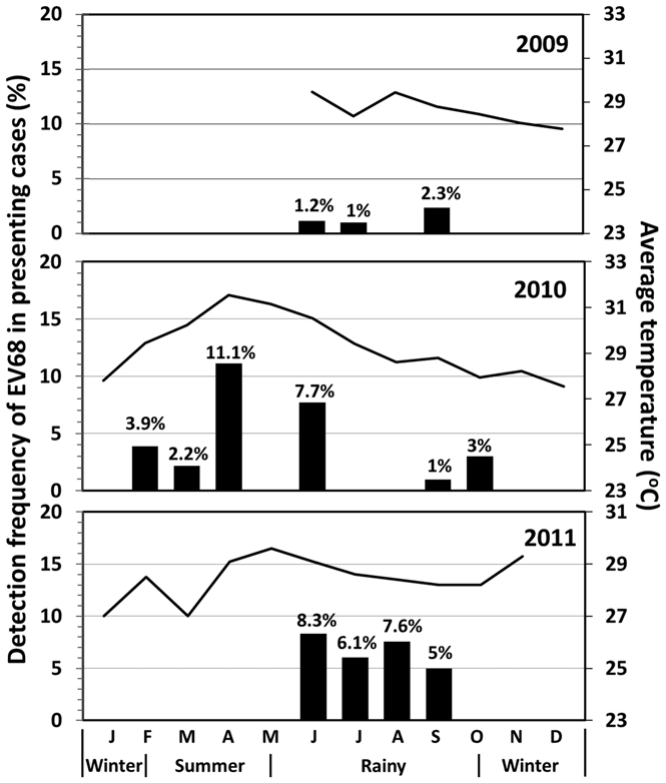
Seasonal distribution of EV68 in Bangkok, Thailand combined from 3 years (2009–2011) during which EV68 cases had been reported. Bar graph shows percentage of pediatric patients with EV68 infection and line graphs indicate monthly average temperatures (degree Celsius (°C)) in Bangkok.

During the first 2 years (February 2006–July 2008), there was no EV68 infection in the sample population (n = 383). Fig 2 provides epidemiological profile of EV68 infection among the patient samples identified in Thailand during 2009–2011. In 2009, 5 EV68 cases were detected in June, July, and September (rainy season) with 0.9% annual prevalence (5/584; all were outpatients). Increased incidence of EV68 infection associated with ARTI was found in 2010 and 2011. In 2010, the virus could be found during a 6-month period with 1.6% annual prevalence (10/611; all were outpatients). During 2011, EV68 infections were investigated only during the rainy season with 4.3% annual prevalence (10/232; 1 was outpatient and 9 were hospitalized patients). Of all EV68 infections detected, 8% were identified from ARTI children admitted to the hospital in summer, 72% during the rainy season, and 20% in winter. Among the meteorological factors, relative humidity was the only factor statistically correlated with the overall number of RV-EV infected patients particularly in 2011 (correlation coefficient (*r_s_*) = 0.61, *p* = 0.04). None of the meteorological factors correlated with the frequency of EV68 detection in Thailand.

### Substitution Rate and Evolutionary Timescale of Circulating EV68 Strains

**Figure 3:**
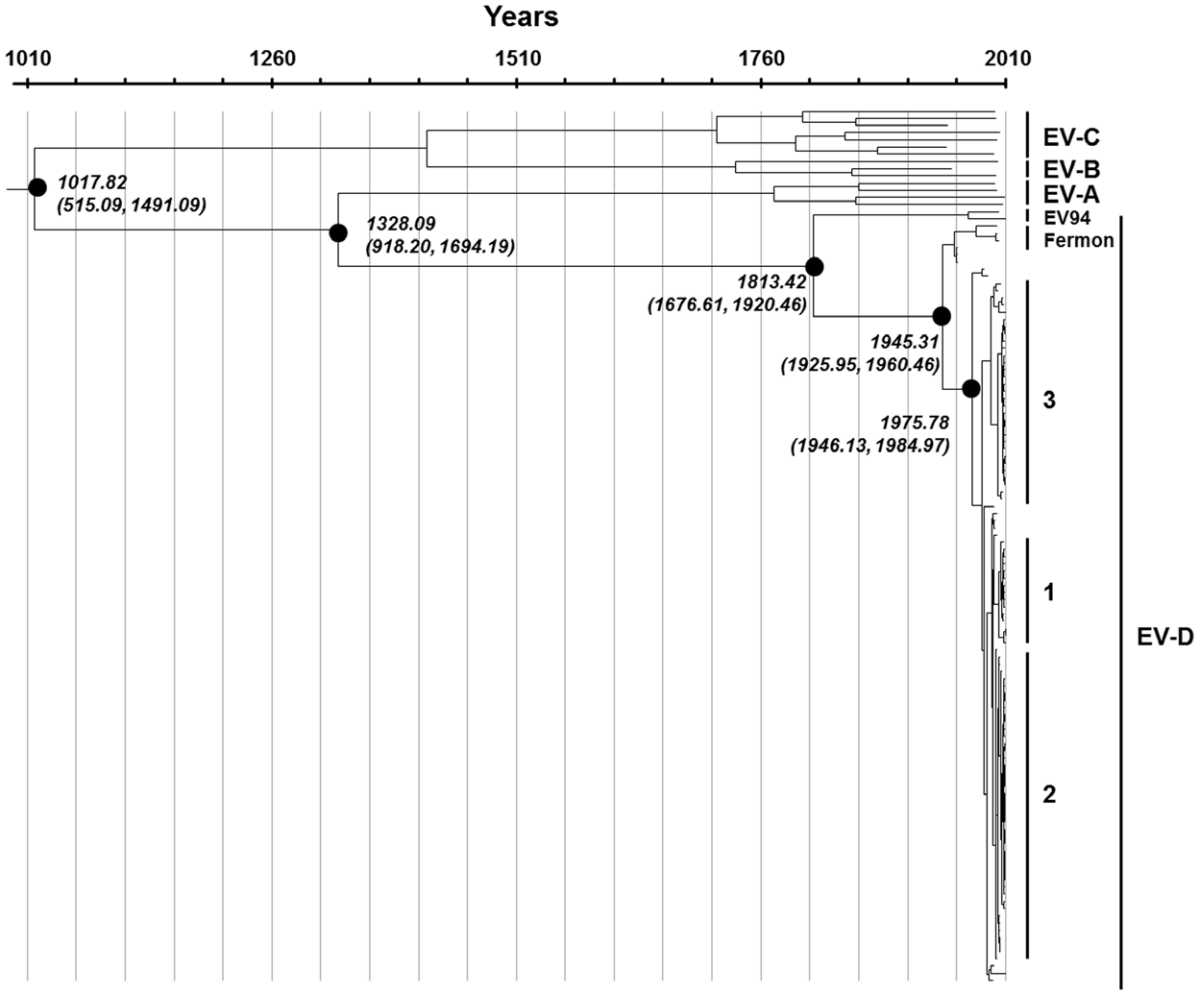
Bayesian time-scale phylogeny of EV68 and other EVs based on the partial VP1 sequence analysis (dataset 1 containing 122 sequences with 723 nt in length). Maximum clade credibility tree obtained with BEAST with a constant size coalescent prior showing lineage splitting events since the most recent common ancestor to the presently circulating EV68 strains. The divergence times correspond to the mean posterior estimate of their ages. For the TMRCA, the correspondent 95% Bayesian credible intervals are shown. Time axis is shown in years and ranges from the TMRCA to the present year.

**Figure 4:**
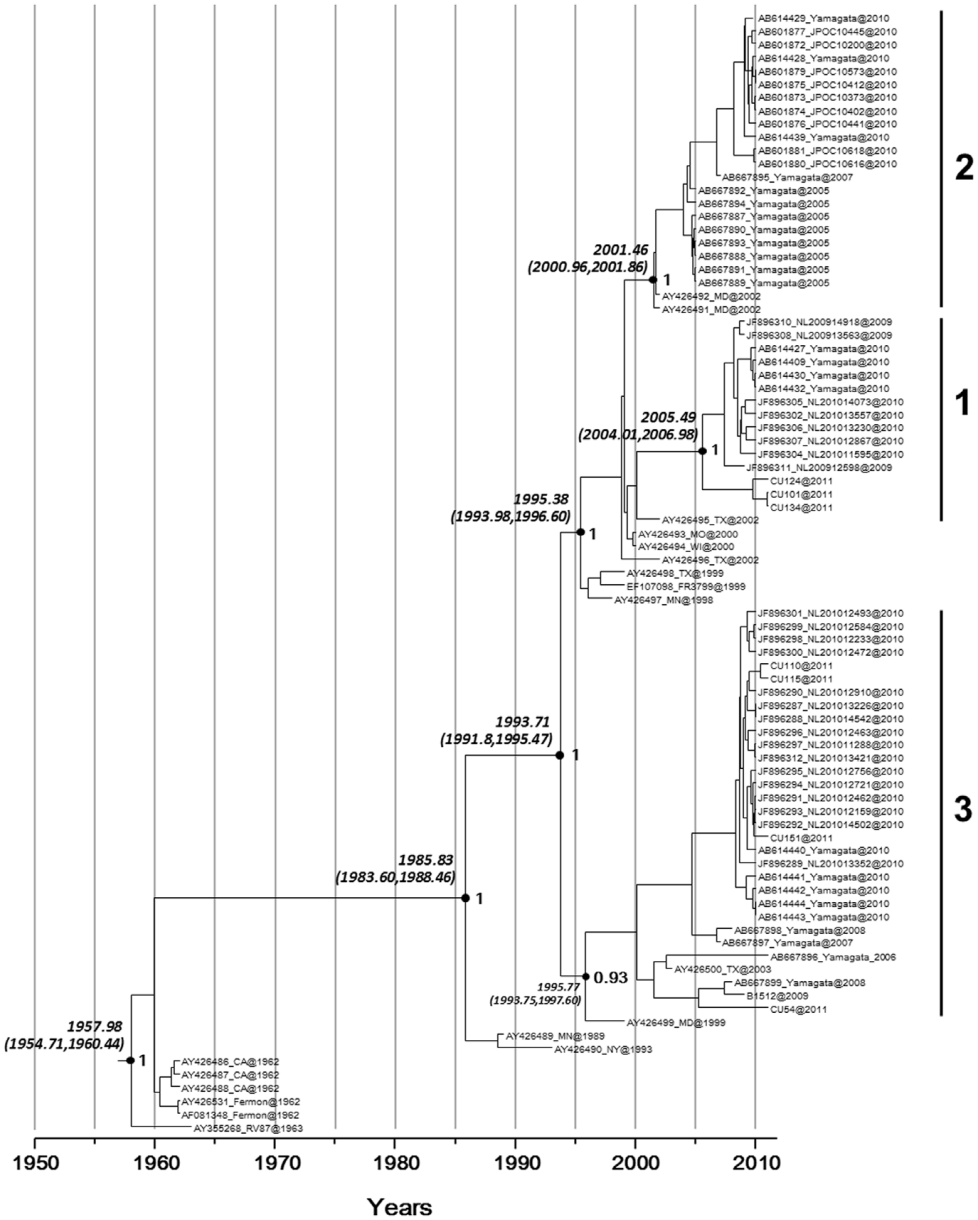
A maximum clade credibility tree from Bayesian timescale phylogenetic analysis of EV68 (dataset 2; n = 106 sequences, length = 723 nt). The posterior probabilities and 95% HPD intervals of the key nodes are depicted above the respective nodes. Three major recent clusters are marked by vertical lines.

Evolutionary rates for VP1 gene were measured as the number of nucleotide substitutions per site per year (s/s/y). By using the Bayesian Markov chain Monte Carlo (MCMC) method, the overall evolutionary rate of the VP1 gene of the entire EV68 sequence dataset (dataset 2) was estimated at 4.93×10^−3^ s/s/y with a 95% HPD limit of 4.01–5.85×10^−3^ s/s/y. The evolutionary rate was lower (3.2×10^−3^ s/s/y with a 95% HPD of 2.27–4.25×10^−3^ s/s/y) when the values were calculated with dataset 1 including a more homologous sampling timescale of all EV species. According to our analysis of the evolutionary timescale of EV68-VP1 sequences (Fig 3), the major bifurcation of the currently circulating EV68 strains from their first isolated prototype (Fermon) occurred 66 years ago (time 1945.31 with (1925.95–1960.46)95% HPD). Based on molecular signature analysis of the BC and DE surface loops of the viral determinant VP1 protein, 3 clusters of EV68 were categorized with the posterior probability (pp) value of 1 for clusters 1 and 2, and pp of 0.93 for cluster 3 (Fig 4). The date when each cluster branched off from the most common recent ancestor (TMRCA) occurred in the 1970s (1975.78, (1946.13–1984.97)95% HPD). Furthermore, EV68 seems to have diverged from their TMRCA with EV species D 198 years ago (1813.42 with (1676.61–1920.46)95% HPD) and with EV species A 680 years ago (1328.09 with (918.20–1694.19)95% HPD).

### Selective Pressure on EV68

**Figure 5:**
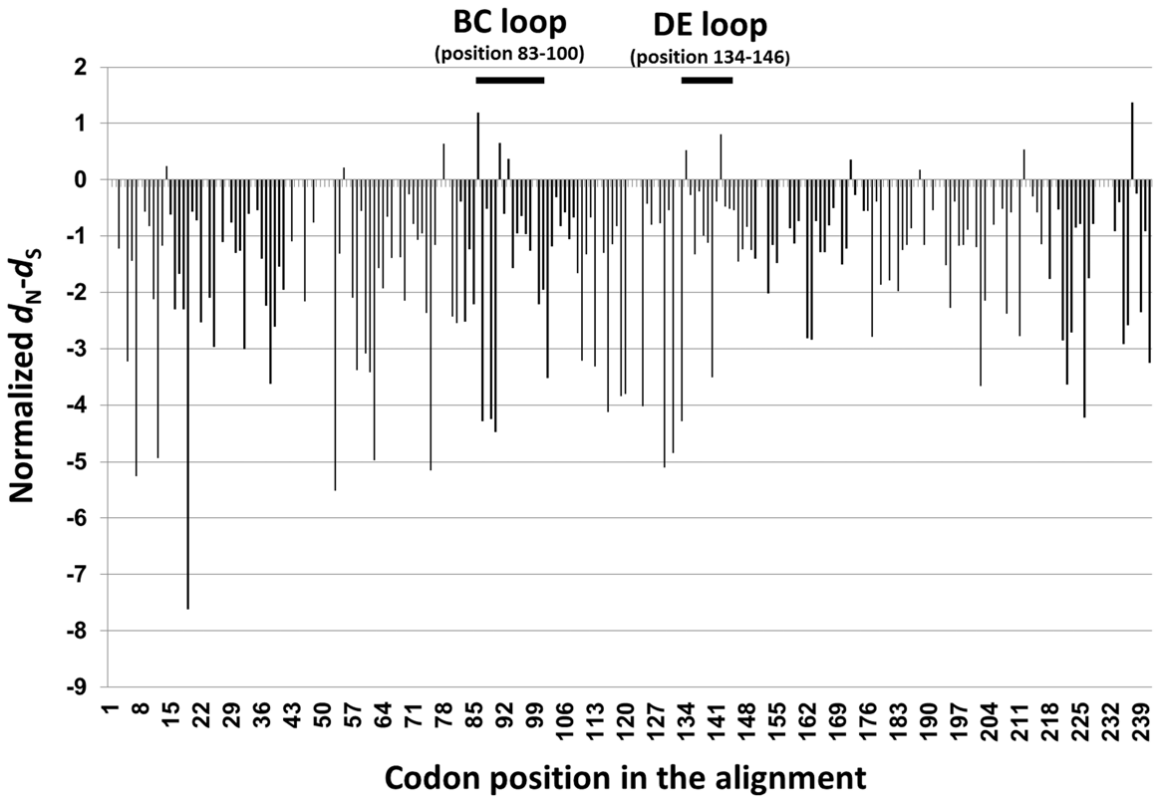
The ratio of nonsynonymous to synonymous substitutions (ω* = d*_N_/*d*_S_) per codon site across the VP1 region of EV68. The residue likely to have evolved under positive selection was recognized when the ω value >1.0. Codon position assigned to this plot was based on the position of the VP1 protein in the EV68 prototype, Fermon (AY426531).

**Figure 6:**
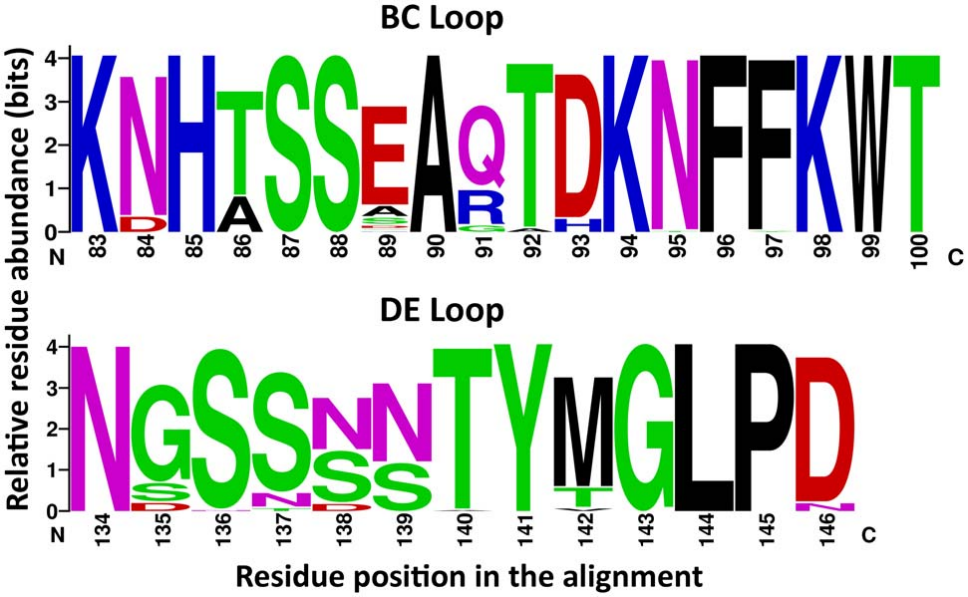
Relative residue abundance at the alignment positions of BC and DE surface-exposed loops. The graphical representation was generated using WebLogo. The height of symbol indicates the relative frequency of the corresponding amino acid at that position. Residue positions are given based on the nucleotide positions of the Fermon strain (AY426531).

To evaluate the adaptive molecular evolution of EV68, overall selective pressure operating on the determinant encoding VP1 was examined by estimating the ratio of non-synonymous (*d*_N_) to synonymous (*d*_S_) substitutions (ω* = d*_N_/*d*_S_) across the lineages on a codon by codon basis. Selective pressure was defined as follows: ω = 1 indicates neutral evolution, ω <1 indicates purifying or negative selection, and ω >1 indicates positive selection. The overall ω value of the VP1 region was 0.091. The majority of amino acid residues in the VP1 region had ω <1 indicating that the amino acids in the antigenic determinant were under purifying selection. To determine whether any specific site or residue of the VP1 region evolved under positive selection, the two rate fixed effects likelihood (FEL) and single likelihood ancestor counting (SLAC) methods were applied. Results from FEL method revealed that codon position 23, 78, 86, 91, 93, 172, 212 and 237 displayed ω>1 without statistical significance. Codon positions 86 and 237 displayed 1.191 and 1.372 normalized *d*_N_–*d*_S_ values, respectively. However, codon position 237 was the only residue that significantly evolved under positive selection (*p* = 0.046). The ω values per codon site across the partial VP1 region of EV68 are shown in Fig 5. The dominant amino-acid compositions at the alignment positions of BC and DE-surface loops of EV68 are showed in Fig 6.

## Discussion

Our longitudinal study investigated the prevalence and clinical characteristics of EV68 infection among Thai children with ARTI who visited various hospitals in Bangkok during 2006–2011. In recent years, with the use of highly sensitive molecular techniques, sporadically detected EV68 virus has been recognized as a re-emerging respiratory pathogen in several countries worldwide since it has been detected with higher frequency in 2008 [8], [12]. EV68 is becoming an increasingly important etiological agent as it was the only pathogen identified in respiratory specimens obtained from fatal cases reported in the retrospective studies of the Philippines and Japan [10], [11]. In the present study, the 25 episodes of EV68 infection associated with ARTI were investigated by phylodynamic relationship analysis, which was performed by evaluating nucleotide sequences of the region encompassing part of the 5′UTR/VP2 and VP1 gene. Our results suggested that children above the age of 5 yrs. seem to be specifically targeted by EV68. Other studies also reported a similar correlation between patient’s age distribution and frequency of EV68 infection [8]–[11], [13], [14]. Despite EV68 infections generally affecting younger children, the association between virus infections and respiratory illnesses in adult patients has also been established [12], [13], [15]. In concurrence with the earlier reports, infection by this virus appears to be an important cause of viral pneumonia in children requiring hospitalization with common clinical presentations of fever, cough, dyspnea, and wheezing [10], [12], [13]. Among children with a history of asthma who experienced more severe asthma attacks, EV68 was the sole etiological agent detected indicating its role as an aggravating factor to severe persistent respiratory disease. Furthermore, a strong association of asthma exacerbation and the virus infection has also been reported [9].

Historically, EV68 has been regarded as an uncommon pathogen associated with respiratory illness. However, until now, the full spectrum of illness caused by the infection of this virus has remained unclear. The involvement of EV68 in central nervous system disease has been reported by various US research teams. Khetsuriani *et al.* discovered the virus from an acute flaccid paralysis case in 2005 [14], while the other group found the virus in cerebrospinal fluid samples taken from fatal meningomyeloencephalitis children in 2011 [16]. Further comparative population-based studies which include other infectious diseases, healthy controls, or adult hospitalized patients should be conducted to elucidate the causal relationship between EV68 infection and disease progression.

In Thailand, no epidemic status of EV68 infection has so far been reported. Our results showed that EV68 probably emerged in Thailand in 2009. Since that time, EV68 infected cases have more frequently been detected during 2010 and, thus far, displayed the highest prevalence in 2011. This finding supported prior observations that the upsurge of EV68 infection has apparently occurred on a global scale since 2010. Distinct seasonal patterns of EV68 infection in ARTI patients were found in Thailand during the study period. Infection rates were higher during the rainy seasons of 2009 and 2011 while no seasonal pattern was apparent in 2010. The variation in EV68-seasonal profile examined in our study was similar to the profiles of respiratory viruses in tropical countries with a higher average temperature throughout the year and less changes in seasonal temperature [17]–[20]. Other studies performed in temperate climates observed seasonality of EV68 in the autumn seasons (Japan 2010 [11], France [21], Netherlands [12], and Italy 2008 [22]). These observations suggested that the seasonal profile of EV68 may vary by season, geographic location, and year.

The VP1 protein is the largest and most exposed surface capsid protein containing the majority of motifs important for interaction with neutralizing antibodies and the cellular receptor required for virus entry. The evolutionary rate, divergence timescale and selective pressure influencing EV68 adaptation should be investigated to provide a better understanding of the genetic diversity and evolutionary relationship of EV68. Results obtained in the present study suggested EV68 could be divided into 3 clusters based on their phylogenetic relationship and the unique molecular signatures within the BC and DE surface loops which was supported by high values of posterior probability. Our suggestion also resembled Meijer’s report [12]. Our study also inferred the rate of evolutionary change for EV68 as 4.93×10^−3^ s/s/y. This value is in accordance with the evolutionary rates of other picornaviruses such as EV71 genotype B (4.2×10^−3 ^s/s/y) [23], [24]. In comparison with other picornavirus members, the rate of evolutionary change estimated for the determinant region of EV68 is faster than the mean evolutionary rate of human parechovirus (2.8×10^−3^ s/s/y) [25], EV71 genotype C (3.4×10^−3 ^s/s/y) [24] and hepatitis A virus (9.8×10^−4^ s/s/y) [26] whereas it is lower than the rate of poliovirus (3×10^−2^ s/s/y) [27], [28]. Consistently, the high evolutionary rate among these viruses relies on several factors including replicase fidelity, highly error prone viral RNA-dependent RNA polymerases resulting in a misincorporation frequency of 1 per 10^3^ to 10^4^ nucleotides [29], rate of transmission, and any synonymous mutations of viral proteins. These factors increase the number of mutations incorporated in viral genomes overtime and prepare the ground for rapid genetic diversification [30]. Moreover, intra- and inter-serotype recombination is also believed to be a large-impact evolutionary mechanism influencing the genetic and antigenic diversity of enterovirus, poliovirus, and other picornaviruses [31]–[40]. Our study also showed that purifying selection plays an important part in shaping the evolution of EV68. This constrained mutation of EV68 can be explained by the limited size and the genetic architecture of the viral genome which is overlapping between structural and functional domains [41]–[43]. With regard to adaptive mutation of EV68 under serological selection, the ω values of specific residues in the VP1 were determined. The result suggested that EV68 tends to escape from stabilizing selection by positively mutating some residues in the antigenic epitope, in particular the residue located on the surface BC and DE loops, resulting in discrimination between the 3 distinct phylogenetic lineages. Differences in the classification of EV68 have been reported by Rahamat-Langendoen *et al* [13]. Two distinct evolutionary lineages comprising old and new clusters were proposed based on phylogenetic analysis of the structural VP4/VP2 and VP1 and year of identification. The old cluster comprised EV68 strains identified in the Netherlands in 2009 and 2010. The new cluster contained all 2010 strains and some of these strains had one amino acid deletion in the VP1. Upon combining these characteristics, the previously defined old and new groups could be reclassified within cluster 1 and 2, respectively.

Despite having performed this study on stored specimens collected since 2006 and utilizing sensitive PCR approaches, some limitations need to be considered. First, it was possible to conclude that ARTI associated with EV68 infection was first introduced to Thailand in 2009 since EV68- infection was not found between 2006 and 2008. Nonetheless, the limited number of specimens towards the end of 2008 and during the first 5 months of 2009 might be an obstacle to our conclusion. Second, although we utilized specimens which we collected from several hospitals located in many different regions, at least 80% of children were Bangkok residents. Therefore, the enrolled children might not be representative of the entire country population. Increased surveillance together with improved laboratory diagnostic techniques will help reveal epidemiology, clinical association, and relationship between the evolutionary rate and the worldwide circulation of this virus. Analyses at the genome level would also be required for a better understanding of the role of selective pressure in this virus evolution.

In conclusion, our study provides additional epidemiological and clinical data on EV68 infection in Thailand indicating worldwide re-emergence of this virus with variations in epidemiological profile. Our data highlighted the potential importance of EV68 as a causative agent of severe respiratory illness which causes hospitalization in children with specific age distribution. We also showed that purifying selection is the predominant evolutionary force acting on the capsid VP1 of EV68 in that some amino acid residues on the surface exposed loops of the viral epitope are positively selected for escape mutation.

## Materials and Methods

### Study Population and Sample Collection

Posterior oropharyngeal and nasal swabs specimens were collected from non-hospitalized patients who had been diagnosed with ILI. Nasopharyngeal aspirations were collected mainly from immunocompromised patients with ALRTI complications and required hospitalizations. A case of ILI was defined according to PAHO/CDC guidelines for influenza surveillance as a progression of fever (>38°C) and either a symptom of cough, sore throat or pharyngitis. Inclusion and exclusion criteria for ALRTI patient enrolment have been described previously [20]. All samples were collected in viral transport media with the addition of antibiotics (2×10^6^ U/L of Penicillin G and 200 mg/l of Streptomycin) and transported within 48 hours to the Center of Excellence in Clinical Virology, Faculty of Medicine, Chulalongkorn University, for routine respiratory virus diagnostic testing. All respiratory specimens were divided into aliquots and stored at −70°C until further tested. Children admitted multiple times with more than one month between visits were considered as separate illness episodes.

### Ethical Consideration

This study was conducted on specimens collected upon conclusion of routine examinations and stored as anonymous. Permission had been granted by the Director of Chulalongkorn King Memorial hospital. Patient identifiers including personal information (name, address) and hospitalization number were removed from these samples to protect patient confidentiality and neither did they appear in any part of document in this study. The research protocol was approved by the Institutional Review Board (IRB number 329/54), Faculty of Medicine, Chulalongkorn University. IRB waived the need for consent because the samples were de-identified.

### Laboratory Diagnosis for Virus-associated ARTI

Viral nucleic acid was extracted from clinical specimens using a 96-well viral nucleic acid extraction kit (RBC Bioscience, Taiwan). cDNA synthesis was achieved with the M-MLV reverse-transcription system (Promega, US) and random hexamer primers according to the manufacturer’s recommendation. Each specimen was tested for common respiratory viruses including RSV-A and RSV-B, Flu-A (pH1N1/2009, seasonal H3 and H1) and Flu-B [44]. For RV-EV screening, semi-nested PCR using a primer set covering the 5′UTR/VP2 region was performed as described elsewhere [20]. The final PCR product of the specimens in which RV implicated was ∼540 nt in length. According to the close relationship between RV and EV genome sequences, samples that contained EV could be identified ∼650 nt. The PCR products were purified using the PCRExtract&GelExtract Mini Kits (5PRIME, Germany) and sequenced bi-directionally by an automated sequencer (First BASE Laboratories, Malaysia). EV68 implicated specimens were confirmed by performing PCR amplifications of the VP1 gene as previously described [13].

### Sequence Analysis

Sequence data for each clinical strain was formatted and assembled by Seqman program of DNASTAR Software (v5.0). To study the distribution and diversity of EV68 identified in this study compared to other EVs, nucleotide sequences of the 5′UTR/VP2 were multiple aligned by using ClustalW implemented in the Bioedit program (v7.0.9). Phylogenetic trees were constructed by neighbor-joining (NJ) method implemented in the MEGA program (v5). The reliability of the NJ tree was estimated using 1000 bootstrap pseudo-replications. Phylogenetic distance among sequences was measured using Kimura’s two-parameter model.

### Meteorological Data

The climate of Thailand is tropical savanna with 3 different seasons including winter (mid-November to mid-February), summer (mid-February to mid-May), and rainy (mid-May to mid-October). Bangkok meteorological data during the study period were provided by the Thailand Meteorology Department (http://www.tmd.go.th/en/). Meteorological data including temperature (degree Celsius (°C)), rainfall (millimeter), and relative humidity (%) were routinely measured at 3-hour intervals in the Bangkok metropolis (standard code 455201) at latitude 13.43.35°N and longitude 100.33.36°E. To determine whether the meteorological factors have an impact on or are associated with EV68 emergences in Thailand, bivariate analysis using 2-tailed Spearman’s coefficient (*r_s_*) were conducted using SPSS software (v17.0) (Chicago, USA).

### Sequence Dataset Preparation

Datasets of the VP1 gene were constructed to perform the Bayesian phylogenetic investigations for estimating the divergence time and determining TMRCA of the recently circulating EV68 lineages. Nucleotide datasets of the VP1 gene were established as follows: dataset 1 was formed by including almost all available EV68-VP1 sequences retrieved from GenBank database by October 31, 2011 and additional nucleotide sequences identified in this study (EV68-TH strains). The homologous sequences of other EVs comprising EV94 for species D (n = 2), CA-A10, EV90 and EV76 as a representative of species A (n = 4) were included in this data set. EV species B and C were assigned as outgroup. Each isolate was labeled with its corresponding year of sampling or isolating. In addition, an alignment of data set 1 containing 122 sequences corresponding to the VP1 gene was constructed (length = 723 nt). Dataset 2 included only EV68-VP1 sequences. The total sequence number of this dataset was 106 sequences with the same length as data set 1.

### Estimated Rate of Evolution and Divergence Time of the Recent Spread of EV68

The rate of substitutions and divergence time of EV68 were estimated by using a Bayesian MCMC approach as implemented in the Bayesian Evolutionary Sampling Tree (BEAST) package (v1.6.2) [45]. The best fit substitution model was chosen by performing a maximum likelihood analysis using the Modeltest package (v3.7) [46]. The best fit model suitable for calculation of nucleotide substitutions was General Time Reversible (GTR)+I (proportion of Invariant site)+G (γ–distribution) model, allowing for nucleotide rates to vary among sites within the capsid coding sequence alignments. Alignments were performed by keeping the first and second codon positions in one partition and the third position in a separate partition as (1+2)+3 codon partitions. In order to accommodate rate variation at the third codon position, the relative rate parameter was separately estimated in each partition.

Relaxed lognormal molecular clocks were employed and followed by allowing substitution rate variations among branches on the trees. Molecular model specification was selected using Bayes Factors and used to quantitatively estimate the growth rate and demographic parameter. The dynamic among study populations was estimated by performing a Bayesian Skyline plot. The Bayesian MCMC chain lengths were 10 million generations with sampling every 10000 generations and discarding 10% of the chain as burn-in. Convergence of the chains was achieved by computational run over a sufficient time with inspection of the MCMC samples using TRACER (v1.4). The resulting tree of each run was summarized using Tree Annotator and the maximum clade credibility tree was visualized with FigTree software (v1.1.2).

### Measurement of Selective Pressure on EV68

The value of ω and the individual site specific selection pressure were measured by using the likelihood based SLAC and FEL contained in the HYPHY package. The database was accessed on the website of Datamonkey interface (http://www.datamonkey.org) [47]. The overall ω value was estimated based on NJ trees under the TrN93 substitution model. The significance level for a positively selected site of SLAC and FEL analyses was accepted at <0.1 (two-tailed binominal distribution). The relative residue abundance within the BC and DE-surface exposed loops were depicted by using WebLogo [48].

### Statistical Analysis

Statistical data comparisons between various factors were analyzed by means of Pearson *χ* ^2^, unpaired T-test, or Fisher’s exact test as appropriate, using SPSS software. All data were considered statistically significant at a *p-*value below 0.05.
